# Neuroblast Distribution after Cortical Impact Is Influenced by White Matter Injury in the Immature Gyrencephalic Brain

**DOI:** 10.3389/fnins.2016.00387

**Published:** 2016-08-23

**Authors:** Sabrina R. Taylor, Colin M. Smith, Kristen L. Keeley, Declan McGuone, Carter P. Dodge, Ann-Christine Duhaime, Beth A. Costine

**Affiliations:** ^1^Department of Physical Medicine and Rehabilitation, Spaulding Rehabilitation HospitalCharlestown, MA, USA; ^2^Brain Trauma Lab, Department of Neurosurgery, Massachusetts General HospitalBoston, MA, USA; ^3^The Office of Chief Medical ExaminerNew York, NY, USA; ^4^Department of Anesthesiology, Dartmouth Medical School, Children's Hospital at DartmouthLebanon, PA, USA; ^5^Department of Neurosurgery, Harvard Medical SchoolBoston, MA, USA

**Keywords:** neurogenesis, neuroplasticity, gyrencephalic, pediatric brain injury, traumatic brain injury, calretinin, interstitial neurons

## Abstract

Cortical contusions are a common type of traumatic brain injury (TBI) in children. Current knowledge of neuroblast response to cortical injury arises primarily from studies utilizing aspiration or cryoinjury in rodents. In infants and children, cortical impact affects both gray and white matter and any neurogenic response may be complicated by the large expanse of white matter between the subventricular zone (SVZ) and the cortex, and the large number of neuroblasts in transit along the major white matter tracts to populate brain regions. Previously, we described an age-dependent increase of neuroblasts in the SVZ in response to cortical impact in the immature gyrencephalic brain. Here, we investigate if neuroblasts target the injury, if white matter injury influences repair efforts, and if postnatal population of brain regions are disrupted. Piglets received a cortical impact to the rostral gyrus cortex or sham surgery at postnatal day (PND) 7, BrdU 2 days prior to (PND 5 and 6) or after injury (PND 7 and 8), and brains were collected at PND 14. Injury did not alter the number of neuroblasts in the white matter between the SVZ and the rostral gyrus. In the gray matter of the injury site, neuroblast density was increased in cavitated lesions, and the number of BrdU^+^ neuroblasts was increased, but comprised less than 1% of all neuroblasts. In the white matter of the injury site, neuroblasts with differentiating morphology were densely arranged along the cavity edge. In a ventral migratory stream, neuroblast density was greater in subjects with a cavitated lesion, indicating that TBI may alter postnatal development of regions supplied by that stream. Cortical impact in the immature gyrencephalic brain produced complicated and variable lesions, increased neuroblast density in cavitated gray matter, resulted in potentially differentiating neuroblasts in the white matter, and may alter the postnatal population of brain regions utilizing a population of neuroblasts that were born prior to PND 5. This platform may be useful to continue to study potential complications of white matter injury and alterations of postnatal population of brain regions, which may contribute to the chronic effects of TBI in children.

## Introduction

Traumatic brain injury (TBI) is the leading cause of death and disability in children, and yet, all promising neuroprotective treatments developed in rodent models have failed in preclinical trials to date (Margulies and Hicks, 2009). One treatment strategy may be the stimulation of innate neuro-regenerative mechanisms to support or replace damaged neurons (Longhi et al., 2004). The effect of TBI on neurogenesis is commonly studied in the hippocampus where TBI has been shown to stimulate neurogenesis in the hippocampal niche in both immature and adult rodent models (Dash et al., 2001; Rola et al., 2006) less studied is the migration of neuroblasts from the subventricular zone (SVZ) to a cortical injury where neuroblasts have been observed to differentiate around the lesion cavity in gray matter in rodent models of TBI (Covey et al., 2010; Saha et al., 2013). In rodent models of stroke, inhibition of neurogenesis has been demonstrated to exacerbate damage (Jin et al., 2010; Marlier et al., 2015). Migration of neuroblasts to the injury and the capacity of neuroblasts to aid in injury repair may be species-specific emphasizing the importance of studying neurogenesis and response to injury in the gyrencephalic brain (Cattaneo and Bonfanti, 2014; Peretto and Bonfanti, 2015). It has yet to be determined if neurogenesis and the targeting of neuroblasts to the injury site is a helpful strategy promoting recovery in the gyrencephalic brain where TBI results in significant white matter injury (Wilde et al., 2006; Wu et al., 2010).

In our large animal model, cortical impact affects both cortical gray matter and cerebral white matter as it deforms 50% of the distance from the cortical gyral crest to the lateral ventricles and thus models blunt impact head injury in children who have deformable skulls and display preferential white matter injury (Duhaime et al., 2000; Missios et al., 2009). We have previously demonstrated an increase in lesion size with increasing age despite injury input parameters scaled for brain growth, and have demonstrated a developmental decrease in the area of the SVZ with increasing age (Costine et al., 2015). In response to scaled cortical impact, the area of neuroblasts in the SVZ increases, but is age-dependent with an increase in piglets developmentally similar to infants (PND 7) and toddlers (PND 30), and no response in piglets developmentally similar to human pre-adolescents (4 months; Costine et al., 2015). The uninjured PND 14 piglet brain has 2 million neuroblasts with a migratory morphology throughout the major white matter tracts (Costine et al., 2015). Neuroblasts are observed to migrate out of the SVZ into the periventricular white matter with the number decreasing with increased age (Sanai et al., 2011; Taylor et al., 2013; Costine et al., 2015).

Blunt impact head trauma during immaturity may not only cause foci of injury, but may also disrupt the normal developmental program of postnatal population of specific brain regions (De Marchis et al., 2004; Sanai et al., 2011; Costine et al., 2015). In normal early postnatal development in gyrencephalic species, large populations of neuroblasts are found in a migratory morphology in the major white matter tracts (Sanai et al., 2011; Costine et al., 2015). The SVZ generates neuroblasts that migrate to distant brain regions via distinct pathways, migratory streams to the olfactory bulbs (where population continues past infancy), prefrontal cortex, and possibly the nucleus accumbens (De Marchis et al., 2004; Sanai et al., 2011). An attempt to repair a traumatic injury in the immature brain may interrupt the postnatal population of specific brain regions resulting in misdirection of migrating neuroblasts from regions undergoing postnatal population or delay progression through damaged white matter. Stalled migration of neuroblasts may potentially result in ectopic foci of neurons, which may act as a substrate for aberrant electrical activity and post-traumatic epilepsy (Taylor et al., 2013; Petit et al., 2014; Irimia and Van Horn, 2015). In addition, mechanical disruption of the structural integrity of gyral white matter tracts following TBI may further impede migration of neuroblasts to the cortical gray matter. In the immature gyrencephalic brain, which utilizes extensive white matter tracts for neuroblast migration during postnatal brain development, disruption of migration patterns might exacerbate complications from TBI (Scharfman and Hen, 2007; Scharfman and McCloskey, 2009; Costine et al., 2015).

Here, we continue our investigation of neuroblasts after cortical impact or sham surgery. We describe for the first time the active postnatal population of the claustrum, which plays a role in the integration of sensory information. The objectives of these experiments were to determine if a cortical impact in the immature gyrencephalic brain results in (1) neuroblasts that target the injury site, (2) disruption of normal postnatal population of the claustrum, and lastly, (3) to determine if neuroblasts in these areas were born before or after cortical impact. Specifically, we administered bromodeoxyuridine (BrdU) just prior to or after TBI or sham surgery in PND 7 piglets and analyzed the brain 7 days after injury. Neuroblasts with or without BrdU were quantified in the white matter tracts leading from the SVZ to the injury, within the injured rostral gyrus, and in a ventral migratory stream that leads to the claustrum, which is normally undergoing active population by neuroblasts at PND 14.

## Materials and methods

### Surgery, BrdU administration, and brain collection

Twenty-nine male, Yorkshire piglets (Earle Parsons and Sons, Inc., Hadley, MA) were housed and fed as previously described after arrival at PND 5 (Costine et al., 2015). Milk replacer was removed overnight prior to surgery and piglets were given a liquid electrolyte solution (BlueLite; TechMix, Inc., Stewart, MN). A total of two piglets died before the end of the experiment: one anesthetic death in a 7 day old piglet likely due to a relative overdose of isoflurane and one piglet was euthanized before the end of the experiment due to illness that developed prior to the start to the experiment (scours, weakness, and weight loss). Four of the sham piglets developed a hematoma at the site of dural incision (discussion below). After careful consideration, these piglets were excluded as the pathology indicated a tissue response to injury that may have interfered with their ability to serve as true shams. A total of 23 piglets were used for analysis. All protocols and procedures were in accordance with the guidelines of the American Veterinary Association and the National Institutes of Health, and were approved by the Animal Care and Use Committee at Massachusetts General Hospital.

Piglets were randomly assigned to a 2 × 2 factorial array experiment with time of BrdU and injury as the main effects resulting in four groups: (1) sham surgery, BrdU before surgery, *n* = 5, (2) sham surgery, BrdU after surgery, *n* = 4, (3) injury, BrdU before surgery, *n* = 7, and (4) injury, BrdU after surgery, *n* = 7. Piglets received a total of 4 injections (Table 1); BrdU (50 mg/kg; IP) or vehicle (saline) was administered as previously described while piglets were lightly anesthetized with isoflurane once per day (Costine et al., 2015). The different time points in administration of BrdU was to compare the number of BrdU^+^ neurons before relative to after injury, in order to identify whether injury itself stimulated the formation of new neurons from the SVZ within the first days after trauma.

**Table 1:** **Timeline and treatment of piglets**.

**Day**	**Day-2**	**Day-1**	**Day 0 Injury or sham**	**Day 1 post-injury**	**Day 2 post-injury**	**Day 3 post-injury**	**Day 4 post-injury**	**Day 5 post-injury**	**Day 6 post-injury**	**Day 7 post-injury**
Age	PND 5	PND 6	PND 7	PND 8	PND 9	PND 10	PND 11	PND 12	PND 13	PND 14
BrdU before	BrdU	BrdU	Vehicle	Vehicle						Brain collection
BrdU after	Vehicle	Vehicle	BrdU	BrdU						

Surgery and anesthesia protocols were employed as previously described (Missios et al., 2009; Costine et al., 2015). Briefly, on PND 7, anesthesia was induced and maintained with isoflurane mixed with room air and piglets were mechanically ventilated. Piglets received buprenorphine (0.005 mg/kg, IM), and heart rate, blood pressure, end tidal CO_2_, oxygen saturation, and body temperature were monitored and maintained within a narrow range (Missios et al., 2009). As previously described, a craniectomy to create a 2 cm burr hole in the skull was performed over the right coronal suture exposing the rostral gyrus, the somatosensory cortex with somatotopy to the snout (Missios et al., 2009). This site was chosen to create a pathologically significant, but clinically silent lesion that we have previously characterized (Duhaime et al., 2000, 2003; Missios et al., 2009). The dura was incised to expose the cortical surface and a spring-loaded indentation device was secured to the skull with screws. The device contained an indentor tip scaled to displace 1% of brain volume in 4 ms (Duhaime et al., 2000; Missios et al., 2009). After injury, the dura was re-approximated and the skin was closed. Sham piglets underwent the surgery at PND 7 without deployment of the indentor. After seven sham surgeries had been performed, it was observed that four sham piglets developed cortical hematomas. It was postulated the incised dura bled onto the cortical surface, and the dura was scored but left intact in the remaining six sham animals. No additional hematomas were observed. Animals were recovered from general anesthesia and weighed daily before euthanasia on day 7 post-injury as an indicator of general health. Average daily gain was not different between sham and injured piglets (0.14 ± 0.2 vs. 0.13 ± 0.2 kg/day; *P* = 0.6).

Seven days after injury (PND 14), piglets were deeply anesthetized and euthanized via exsanguination by transcardial perfusion with 0.9% saline followed by 10% buffered formalin as previously described (Missios et al., 2009; Costine et al., 2015). The brain was removed, post-fixed overnight at 4°C, and coronally sliced into 5 mm blocks starting at the anterior edge of the rostral gyrus. The three adjacent 5 mm coronal blocks containing the injury site and SVZ were post-fixed in 10% buffered formalin overnight at 4°C then cryoprotected in 30% sucrose in phosphate buffered saline (PBS) over 2–3 days at 4°C. Blocks were embedded in optimum cutting temperature compound (OCT, Tissue-Tek®), frozen, and stored at −80°C. Frozen sections were made (50 μm) and every other section was mounted on poly-L-lysine-coated microscope slides (Superfrost® Plus, Fisherbrand®, Fisher Scientific, Pittsburgh, PA), dried, and stored at −80°C.

### Histologic analysis

In order to ascertain the presence and severity of injury, two 10 μm sections from each piglet (one from each hemisphere) were obtained just caudal to the impact site. The sections were stained with Luxol-fast-blue/hematoxylin-eosin (LH&E) and were analyzed by a neuropathologist (DM) blinded to injury and hemisphere for contusional injury, hemorrhage, necrosis, and acute and chronic inflammation.

Immunofluorescence was used to assess the effect of trauma on cell types indicative of neurogenesis (DCX^+^, BrdU^+^, and DCX^+^/BrdU^+^ cells) or markers of early neuronal differentiation (calretinin) or mature neurons (NeuN) as previously described (Costine et al., 2015; Sarnat, 2015). Between all steps, sections were washed three times in 0.1 M tris-buffered saline (TBS; 1 M Tris-HCl, 1.5 M NaCl; pH 7.5). Mounted tissue was rehydrated in TBS followed by permeabilization in 1% Triton X-100 (ICN Biomedicals, Aurora, OH) in TBS (TBST) for 20 min. Sections were placed in 10 mM citric acid buffer (pH 6.0) in a pressure cooker (DAKO, Carpinteria, CA) reaching 120°C for 10 min and 90°C for 20 min for concurrent antigen retrieval and DNA denaturation (Tang et al., 2007). The tissue was allowed to equilibrate to room temperature for 30 min before blocking with 5% donkey serum/TBST (TBSTD, ab7475, Abcam, Cambridge, MA) for 30 min. Sections were incubated in various combinations of the following primary antibodies: goat anti-human DCX (sc-8066, Santa Cruz Biotechnology, Santa Cruz, CA; 1:100), mouse anti-BrdU (ab8152, Abcam; 1:75), rabbit anti-calretinin (ab702, Abcam; 1:100), mouse anti-CD68 (M0876, Dako; 1:100), anti-mouse NeuN (MAB377; Millipore;1:100) and were diluted in TBSTD overnight at 4°C. We previously determined that this DCX antibody does not co-localize with markers of astrocytes nor microglia (Taylor et al., 2013). After washing the primary antibody, sections were incubated in Alexa Fluor 488 donkey anti-goat (for DCX) and Alexa Fluor 568 donkey anti-mouse (for BrdU) or Alexa Fluor 568 donkey anti-rabbit (for calretinin;1:200, Invitrogen, Carlsbad, CA) or Alexa Fluor 488 donkey-anti-mouse for CD68 or NeuN for 2 h at room temperature protected from light. Sections were then coated with Vectashield Mounting Medium with 4′,6-diamidino-2-phenylindole (DAPI) to detect all nuclei (Vector Laboratories, Inc., Burlingame, CA) or were incubated for 15 min in 0.5 μM TO-PRO®-3 Iodide in PBS (TOPRO; Thermofisher Scientific, Cambridge, MA) and cover slipped. Negative control sections excluded primary antibodies. In the absence of primary antibody, some blood vessel structures were observed. In the center of the lesion cavity, auto-fluorescence of red blood cells and perhaps other cellular debris was observed and was not imaged. Black holes in images are artifact from freezing that was primarily observed in gray matter.

### Stereological quantification of cell types in the white matter tract leading from the SVZ to the rostral gyrus

**Figure 1:**
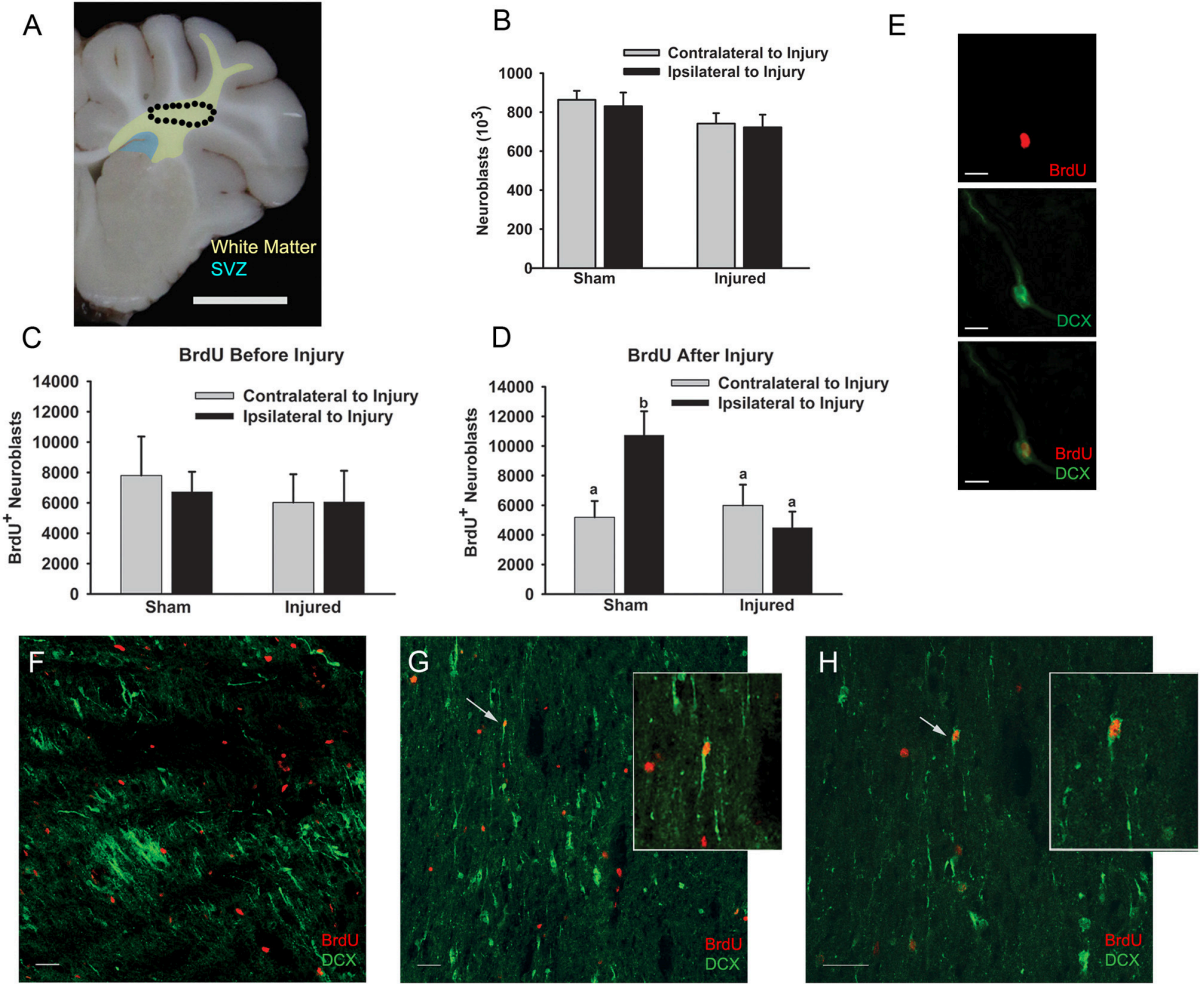
**Cortical impact did not alter the number of neuroblasts or BrdU^+^ neuroblasts in the white matter tracts leading to the injury**. Piglets received cortical impact or sham surgeries on PND 7 with BrdU administered prior to injury or after injury, and the brains were collected at PND 14. **(A)** Schematic illustrating the SVZ (blue) and the white matter where neuroblasts were quantified (yellow) including the corpus callosum, centrum semiovale (circle), and the white matter of the rostral gyrus. The number of neuroblasts **(B)** and the number of BrdU^+^ neuroblasts when BrdU was given before **(C)** or after **(D)** injury was not greater in injured vs. sham piglets as determined with two-way ANOVA followed by Fisher's LSD *post-hoc* analyses. **(D)** However, the number of BrdU^+^ neuroblasts in the ipsilateral hemisphere of sham piglets was greater than the contralateral hemisphere and both hemispheres of injured piglets (^a, b^Means ± SEM with different letters differ, *P* < 0.05). **(E)** High power photomicrograph demonstrating DCX (green) and BrdU (red) co-labeling in a neuroblast with a migratory morphology. **(F)** Neuroblasts and BrdU^+^ cells in the corpus collosum of sham piglets. **(G,H)** Neuroblasts and BrdU^+^ cells in the white matter of the rostral gyrus of sham piglets with arrows indicating BrdU^+^ neuroblasts. Inset: Increased magnification of BrdU^+^ neuroblasts. Scale bars: **(A)** = 1 cm; **(F**–**H)** = 20 μm; **(E)** = 10 μm.

The total numbers of DCX^+^ (“neuroblasts”), BrdU^+^(“BrdU^+^ cells”), and DCX^+^/BrdU^+^ cells (“BrdU^+^ neuroblasts”) in the white matter from the SVZ to the site of injury (Figure 1A) were quantified with the optical fractionator method (Francis et al., 2006). A Nikon TE-2000 microscope with a motorized XYZ stage was used for quantification. Quantification was completed on 13–18 sections per animal (6–9 sections per hemisphere), which were positioned every 1200 μm after a random start within the first 12 sections. Sections were coded and identifiers removed on the slide for analysis. Briefly, the Stereo Investigator software was used to draw a contour around the white matter from the abventricular SVZ to the impact site on the cortex including the centrum semiovale (the white matter under the gray matter in the cerebrum; Figure 1A) using a 2x objective. Once the region of interest had been outlined, quantification of cells was carried out using a 20x objective. The sampling scheme was as follows: sampling grid: 859 × 1255 μm (with random orientation); counting frame: 200 × 200 μm; dissector height: 30 μm; and guard zones: 5 μm. Every DCX^+^, BrdU^+^, and DCX^+^/BrdU^+^ cell was tagged with a specific marker and tracked in the Stereo Investigator section manager to avoid over-counting. When the specific tags for DCX^+^ and BrdU^+^ cells were proximally located, co-localization was confirmed at 40x before tagging the cell with a third tag for DCX^+^/BrdU^+^ cells. The area of the region of interest was determined using Stereo Investigator, and the total numbers (N) of DCX^+^, BrdU^+^, and DCX^+^/BrdU^+^ cells were then calculated with the optical fractionator formula: $N\;=\;\frac{1}{ssf}\;\times \;\frac{1}{asf}\;\times \;\frac{1}{hsf}\;\times \;\sum Q^{-}$. For this experiment the section sampling fraction (ssf) was $\frac{1}{24}$, the area sampling fraction (asf) was $\frac{1}{27}$, and the height sampling fraction (hsf) was ~$\frac{30}{36}$. As before, ∑*Q*^−^ was the total count of particles sampled for the entire white matter area.

DCX^+^ cells were characterized as neuroblasts if they had an elongated morphology with uni—or bi-polar processes to indicate migration (“neuroblasts”). Cells were considered BrdU^+^ if they had nuclei, visualized with DAPI, which were positive for BrdU (“BrdU^+^ cells,” not co-localized with DCX). Neuroblasts were quantified as newborn cells if the nucleus was positive for BrdU with a surrounding cytoplasm positive for DCX (“BrdU^+^ neuroblasts,” Figures 1E–H).

### Quantification of cells in region of the injury, a ventral migratory stream, and claustrum

**Figure 2:**
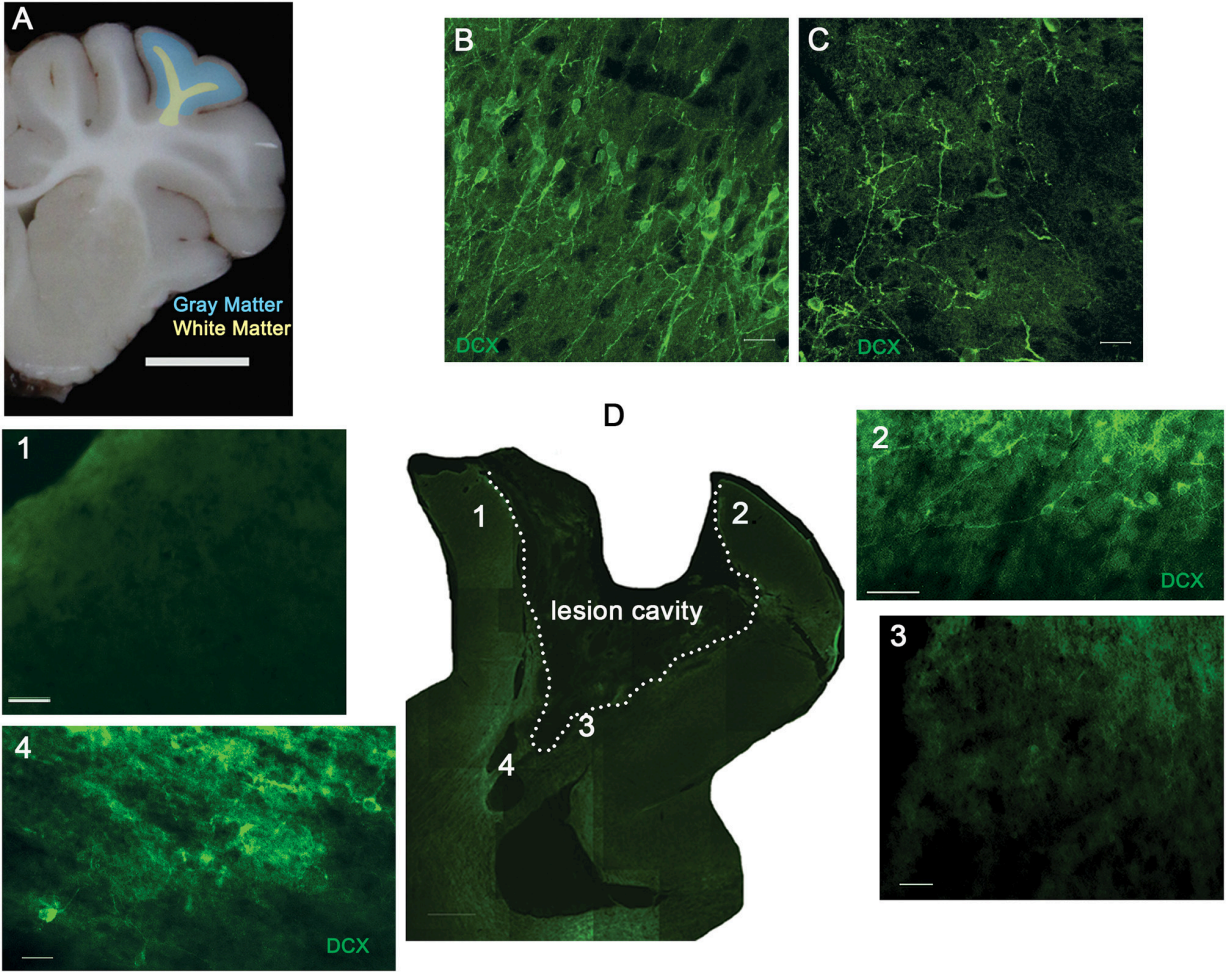
**The density of neuroblasts throughout the lesion is variable**. Piglets received cortical impact or sham surgery on PND 7, and the brain was collected at PND 14. **(A)** Schematic illustrating the location of the cortical impact and resulting lesion in the rostral gyrus gray matter (blue) and rostral gyrus white matter (yellow). The second layer of the rostral gyrus contained pyramidal neurons positive for DCX (green) in sham piglets **(B)** that was disrupted in injured piglets **(C)**. **(D)** Low power view of the injured rostral gyrus with a lesion cavity outlined (broken line, “cavity^+^”) with higher power images demonstrating a lack of neuroblasts in the gray matter **(1)**, disruption of the second layer **(2)**, lack of neuroblasts in the white matter adjacent to the lesion cavity **(3)**, and increased neuroblast density in the white matter at the base of the gyrus **(4)**. Scale bars: **(A)** = 1 cm; **(D)** = 200 μm; **(D1**–**D4)** = 50 μm; **(B**,**C)** = 20 μm.

To further investigate the region of the injury and other brain regions where injury may affect neuroblast density, microphotographs were analyzed as a more time efficient method than stereology. The other advantage was that microphotographs could be quantified by multiple workers blinded to the treatment (without having access to view the tissue section on the microscope), and easily audited by multiple workers. Images were obtained using a 20x objective with the microscope and camera (described above) of four fields in 2 sections from each piglet for the following regions: rostral gyrus gray matter, rostral gyrus white matter, a ventral migratory stream in the external capsule, and the claustrum. Sections were selected from the contralateral hemisphere of sham piglets or the ipsilateral hemisphere of injured piglets. To ensure neuroblasts were quantified in injured sections, we required evidence of injury in the section analyzed that were binned into 2 categories: (1) large cavitation through the gray matter extending into the gyral white matter and sometimes deeper into the subcortical white matter: “cavity^+^ lesion,” (2) disruption in the gray matter of the gyral crest surface at the impact site only: “cavity^−^ lesion.” One injured piglet was excluded as an area of injury was not visible on immunofluorescence in multiple sections evaluated (though an injury was observed for that piglet on tissue stained with LH&E). To select the area for imaging in the rostral gyrus, the entire gyrus was evaluated for areas of high neuroblast density, and 1–2 photos were obtained for gray or white matter. Thereafter, images were obtained for gray matter in the dorsal portion of the gyrus dorsal to the termination of the gyral white matter and mid-gyrus to the right and left of gyral white matter. White matter images were obtained at evenly spaced distances along the gyral white matter spanning the tract. Cortical layer II pyramidal neurons were DCX^+^ in all piglets and this layer was often disrupted in injured piglets and was, therefore, not included in imaging for cell quantification (Figures 2B,C). In images removed of subject and treatment identifiers, the numbers of DCX (“neuroblasts”), BrdU (“BrdU^+^ cells”), and DCX/BrdU cells (“BrdU^+^ neuroblasts”) were quantified using the “Cell Counter” plugin of ImageJ (National Institutes of Health, Bethesda, MD). The eight fields per region per piglet per cell type (BrdU^+^, BrdU^+^ neuroblasts, neuroblasts) were averaged and the maximum count was identified.

### Preliminary analysis of neuroblast morphology in regions of injured white matter

To further characterize the morphology of neuroblasts according to the region within the injured white matter, photomicrographs of the white matter were analyzed in a subset of piglets with cavity^+^ lesions (*n* = 2). Using the “Simple Neurite Tracer” plugin in ImageJ, neuroblasts, as detected with DCX, were analyzed in single plane or z stack photomicrographs in the injured white matter either adjacent to the cavity (*n* = 244 neuroblasts in three photomicrographs; **Figure 4**) or in the injured white matter at a distance from the cavity (*n* = 143 neuroblasts in three photomicrographs). The total number of branches was recorded per neuroblast. The mean number of branches per neuroblasts in each injury location and the percentage of unipolar (no branches), bipolar (1 branch off the neuroblast), and multipolar neuroblasts (2 or more branches off the neuroblast) per injury location were determined. For example, the neuroblast in Figure 1E is considered “unipolar” with only the main neuroblast. The neuroblast in **Figure 4E** was considered “multipolar” with three branches + the main neuroblast.

### Statistical analysis

For the stereological investigation of white matter tracts, the main effects of the timing of BrdU administration, injury, and their interaction on cell numbers (neuroblasts, BrdU^+^ cells, and BrdU^+^ neuroblasts) were analyzed with two-way analysis of variance (ANOVA) followed by Fisher's Least Significant Difference (Fisher's LSD) *post-hoc* analyses. For quantification of cells at the injury site, no differences in the time of BrdU administration on BrdU^+^ neuroblasts were detected via a Student's *t*-Test, therefore, these groups were combined. The differences in the density of BrdU^+^ cells, neuroblasts, and BrdU^+^ neuroblasts in the rostral gyrus white matter, rostral gyrus gray matter, external capsule, and claustrum were compared in injured vs. sham piglets using unpaired Student's *t*-Tests. The difference in the number of neuroblast branches per neuroblast in the injured white matter at the cavity edge vs. at a distance from the cavity edge in the injured white matter were tested with an unpaired Student's *t*-Test. The main effect of injury on the maximum density of neuroblasts in piglets binned for lesion type (“cavity^+^ lesion” or “cavity^−^ lesion”) were determined via a one-way ANOVA followed by Tukey-Kramer Multiple Comparisons Test. All values were expressed as means ± SEM. *P* < 0.05 were considered significant. *P* ≤ 0.08 were considered as a tendency to be significant. Statistical analyses were performed using SPSS Statistics software version 20 (IBM, Armonk, NY) or Prism® version 6.03 (GraphPad, San Diego, CA).

## Results

### Effect of cortical impact on neuroblast quantity in the cerebral white matter leading to the injured rostral gyrus

In this immature gyrencephalic species, neuroblasts appear to travel along the extensive white matter tracts from the SVZ to the cortical gyri. To determine if a greater number of neuroblasts were in the white matter between the SVZ and the injured rostral gyrus compared to sham injured animals, neuroblasts were quantified within the corpus callosum adjacent to the SVZ, centrum semiovale, and white matter of the rostral gyrus ipsilateral and contralateral to injury (Figures 1A, E–H) in injured and sham piglets. BrdU was administered daily for 2 days prior to injury (PND 5 and 6) or after injury (PND 7 and 8) to determine the timing of the birth of the neuroblasts in the white matter 7 days after injury (PND 14; Table 1). The numbers of BrdU^+^ cells were not different if BrdU was given before or after sham surgery (4.6 × 10^6^ ± 1.0 × 10^6^ vs. 3.51 × 10^6^ ± 0.49 × 10^6^) or injury (3.8 × 10^6^ ± 1.4 × 10^6^ vs. 3.3 × 10^6^±8.5 × 10^6^) indicating that there was no effect of piglet age at the time of BrdU injection (5–6 PND vs. 8–9 PND) on proliferation and that an additional proliferative effect on cell genesis due to the injury was not observed in the white matter tracts leading to the injury. Neither the number of neuroblasts nor the number of BrdU^+^ neuroblasts when BrdU was given before injury differed among injured vs. sham piglets (Figures 1B,C). Unexpectedly, the number of BrdU^+^ neuroblasts in the ipsilateral hemisphere of sham piglets was greater than the contralateral and both hemispheres of injured piglets when BrdU was administered after injury (main effect of hemisphere, *P* = 0.32, main effect of injury, *P* = 0.051, interaction, *P* = 0.027, Fisher's LSD, *P* < 0.05; Figure 1D).

### Cortical impact produces different patterns of tissue damage in the piglet

The rostral gyrus of all injured piglets demonstrated microscopic evidence of apoptosis, hemorrhage, necrosis, acute or chronic inflammation, reactive astrocytes, microgliosis, macrophages, and axonal swellings (data not shown). The response to impact resulted in variable injury patterns, therefore, data was analyzed among all lesion types and also binned into two categories: (1) large cavitation through the gray matter extending through the cortical ribbon into the gyral white matter and sometimes extending into the centrum semiovale: “cavity^+^ lesion,” (2) disruption to the impact site of the cortical gray matter only: “cavity^−^ lesion.” These categories reflect the extent of tissue disruption and magnitude of the visible lesion. We did not observe lesions extending to the SVZ in this age of piglets.

### Neuroblast density is increased at the injury, but the density is highly variable

Because there was no effect of injury on the total number of neuroblasts or BrdU^+^ neuroblasts in the white matter leading from the SVZ to the injury, we sought to determine if neuroblasts were concentrated at the site of injury in the rostral gyrus. Both the gray matter and white matter of the rostral gyrus was analyzed with the white matter being a subset of the entire white matter tract analyzed above (Figure 2A). Not surprisingly, neurons in layer II of the rostral gyrus were DCX^+^ and displayed a differentiated, mature neuronal morphology (Figure 2B). This layer was disrupted by the injury and was not included in the analysis (Figures 2C,D2). Neuroblast density was variable in the gray matter and white matter of the injured rostral gyrus. In cavity^+^ lesions (Figure 2D), the gray matter of the dorsal rostral gyrus was devoid of neuroblasts (DCX was below the level of detection) in gray matter that remained intact (Figures 2D1,D3) while pockets of dense neuroblasts were found at ventral portions of the gyrus in the white matter adjacent to lesion cavities (Figures 2D4, 4B–J).

**Figure 3:**
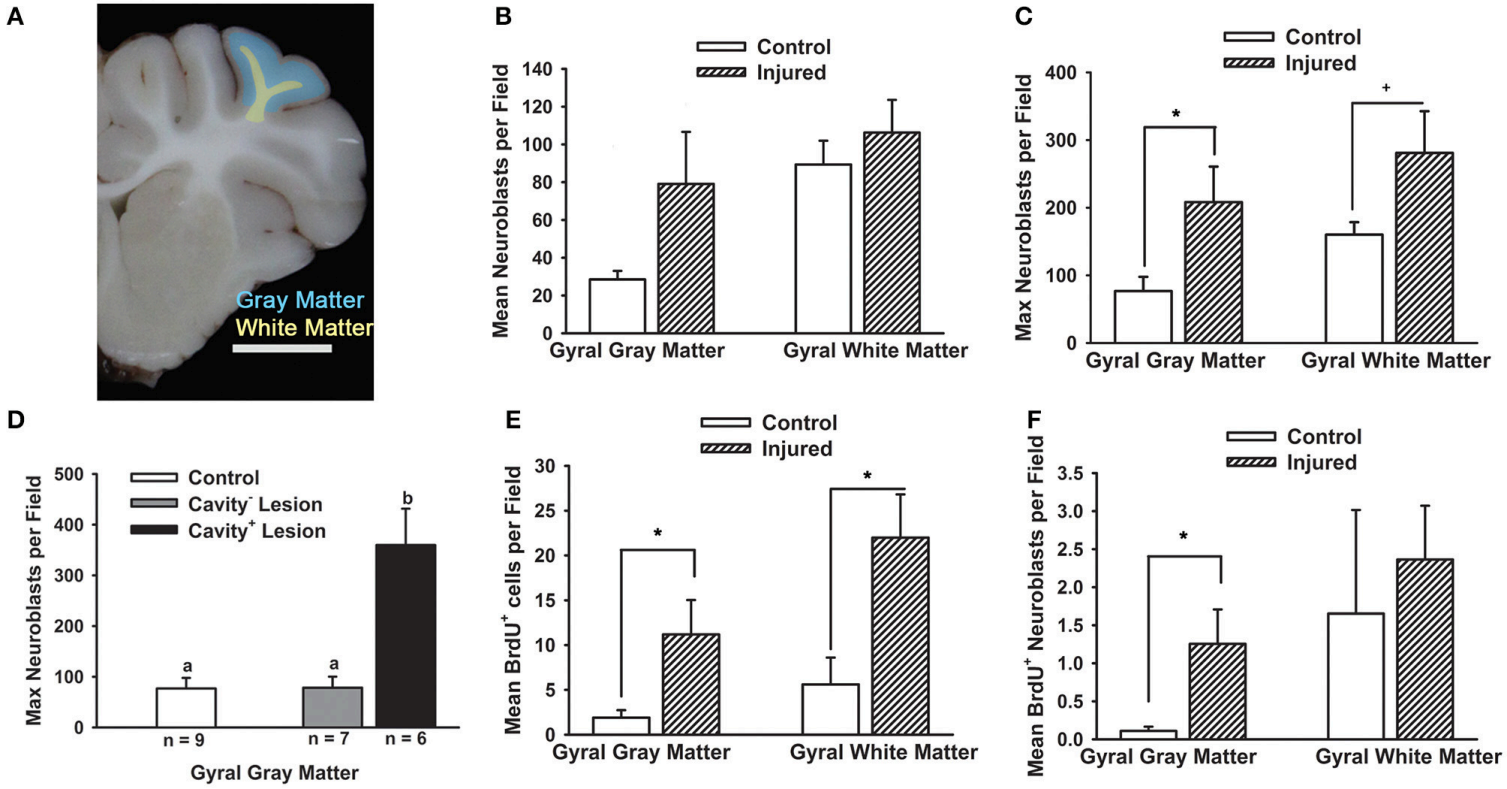
**Cortical impact increases the density of neuroblasts in the gray matter and white matter dependent on lesion type**. Piglets received cortical impact or sham surgery on PND 7 and the brain was collected at PND 14. The mean density of neuroblasts (average of eight fields per region per piglet) and max density of neuroblasts (field with the greatest density) in each region was compared in injured vs. sham piglets via Student's *t*-Tests. **(A)** Schematic illustrating the location of the cortical impact and resulting lesion in the rostral gyrus gray matter (blue) and rostral gyrus white matter (yellow). **(B)** The mean neuroblast density was not greater (*P* = 0.09) in injured vs. sham piglets. **(C)** The maximum neuroblast density was greater (^*^P < 0.05) in the gray matter and tended to be greater (^+^*P* = 0.08) in the white matter of the rostral gyrus. **(D)** Piglets were binned into categories of lesion type based on pattern and extent, and neuroblast density was compared among groups via a one way ANOVA followed by Tukey-Kramer Multiple Comparisons Test. Injured piglets with a cavity^+^ lesion had a greater maximum density of neuroblasts than piglets with “cavity^−^” lesions (^a, b^Means ± SEM with different letters differ, *P* < 0.05). **(E)** The mean number of BrdU^+^ cells per field was greater in the gray and white matter of injured vs. sham piglets (^*^P < 0.05). **(F)** The mean number of BrdU^+^ neuroblasts per field was greater in injured rostral gyrus gray matter (^*^P < 0.05), though the number is extremely low.

The mean neuroblast density was approximately two-fold greater in the gray matter of the rostral gyrus among all lesion types in injured vs. sham piglets, but was not significant (*P* = 0.09; Figures 3A,B). Due to within-subject variability of neuroblast distribution within each subject, the maximum density per field per piglet was also identified. Maximum neuroblast density was greater (*P* < 0.05) in the gray matter and tended to be greater (*P* = 0.08) in the white matter of the rostral gyrus of injured piglets vs. sham piglets (Figure 3C). When binned into lesion category, the increase in maximum neuroblast density was due to an increase in cavity^+^ lesions only (ANOVA *P* = 0.0029; Tukey-Cramer: cavity^+^ lesion vs. sham *P* < 0.01, Tukey-Cramer: cavity^+^ lesion vs. cavity^−^ lesion *P* < 0.05; Figure 3D). As expected, injury increased the number of BrdU^+^ cells in the rostral gyrus gray matter and white matter (*P* < 0.05; Figure 3E), of which only 10% were neuroblasts (Figure 3F). The number of neuroblasts that were BrdU^+^ did not differ when BrdU was administered before or after injury and these groups were therefore combined for analyses. The number of BrdU^+^ neuroblasts was greater in injured gray matter (*P* < 0.05), but not gyral white matter (Figure 3F). Only 1% of neuroblasts at the injury were BrdU^+^ (Figures 3B,F).

### Apparent differentiation of neuroblasts in the injured white matter in some cavity^+^ lesions

**Figure 4:**
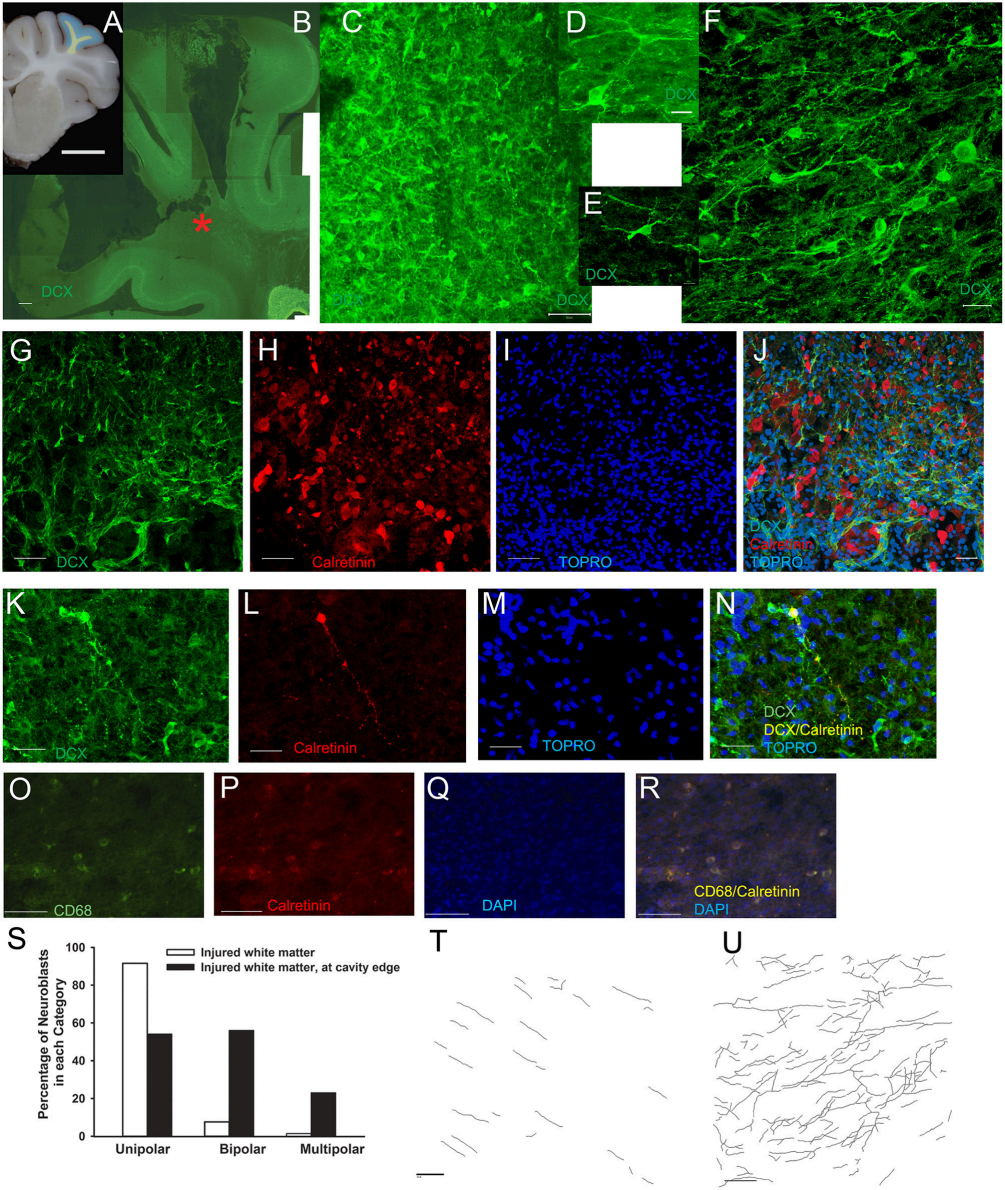
**Neuroblasts in injured white matter appear to be differentiating**. In cavity^+^ lesions, neuroblasts along the edge of the cavity were observed have a differentiating morphology. The white matter of the rostral gyrus was examined (yellow; **A**), and neuroblasts were densely arranged at the edge of the cavity at the ventral portion of the gyral white matter (red star; **B**). **(C**–**F)** Higher power images of the dense meshwork of neuroblasts in the injured rostral gyrus white matter indicated by the red star in **(B)**. **(D,E)** Neuroblasts (green) with a multipolar, differentiating morphology with 4–5 neurites at the cavity edge intermixed with calretinin^+^ neurons (red). **(K–N)** A few neuroblasts (green) in the injured white matter were found to co-localize with calretinin (red; yellow when co-localized). **(O–R)** In the injured white matter (not on the cavity edge), small groups of small, round macrophages were faintly positive for CD68 (green) and faintly positive for calretinin (red; yellow when co-localized). **(S)** At the cavity edge, neuroblasts had a greater number of neurites (*P* < 0.001) with a larger percentage of bipolar and multipolar neuroblasts than in the injured white matter not adjacent to the cavity edge. **(T,U)** Maximum projection of neurite tracing in injured white matter distant from the cavity edge **(T)** and at the cavity edge **(U)** in injured piglets with a cavity^+^ lesion. Scale bars: **(A)** = 1 cm; **(B)** = 500 μm; **(K–R)** = 100 μm; **(C,G–J,T,U)** = 50 μm; **(F)** = 20 μm; **(D**,**E)** = 10 μm.

Dense meshworks of neuroblasts displaying a differentiating morphology were observed in some ventral portions along the edge of the lesion cavity in the white matter (Figures 4A–F). In a preliminary analysis of injured white matter, neuroblasts adjacent to the cavity edge had a greater number of neurites than neuroblasts at a distance away from the cavity edge (2.05 ± 0.1 vs. 1.10 ± 0.03, *P* < 0.001; 1 main branch only = unipolar neuroblasts, 2 = bipolar neuroblasts consisting of the main branch + 1 neurite, 3 + = multipolar neuroblasts; *N* = 387 neuroblasts; Figures 4S–U). In injured white matter at a distance from the cavity, 91% of neuroblasts were unipolar, 7.6% were bipolar, and only 1.4% were multipolar (Figure 4S). The majority of neuroblasts in injured white matter at a distance from the cavity appeared to be in a simple migratory phenotype (Figure 4T). The maximum number of neurites observed to branch off of multipolar neuroblasts in this region was 2 neurites. In contrast, in the region adjacent to the cavity in the white matter, 52.5% were unipolar, 23% were bipolar, and 24.5% were multipolar (Figure 4S). Of multipolar neuroblasts adjacent to the cavity, 30% had 5 or more branches having up to 10 neurites. Often, neurites from multipolar neuroblasts were observed to be in close proximity to other neurites from other multipolar neuroblasts (Figure 4U). The white matter adjacent the white cavity had a mixture of neuroblasts in a migratory and a differentiating morphology.

To further investigate these neurons that appear to be sprouting neurites, we probed calretinin, a marker of GABAergic neurons expressed early in differentiation. As expected, calretinin^+^ neuroblasts were observed throughout the gray matter, but were also observed at the cavity edge intermixed with DCX^+^ neuroblasts (Figures 4G–J). We rarely observed co-labeling of calretinin and DCX in the same neuron (Figures 4K–N). Also in the injured white matter, but not on the cavity edge were small, round macrophages that were CD68^+^ (Figures 4O–R).

### Cortical impact does not disrupt postnatal population of the claustrum

**Figure 5:**
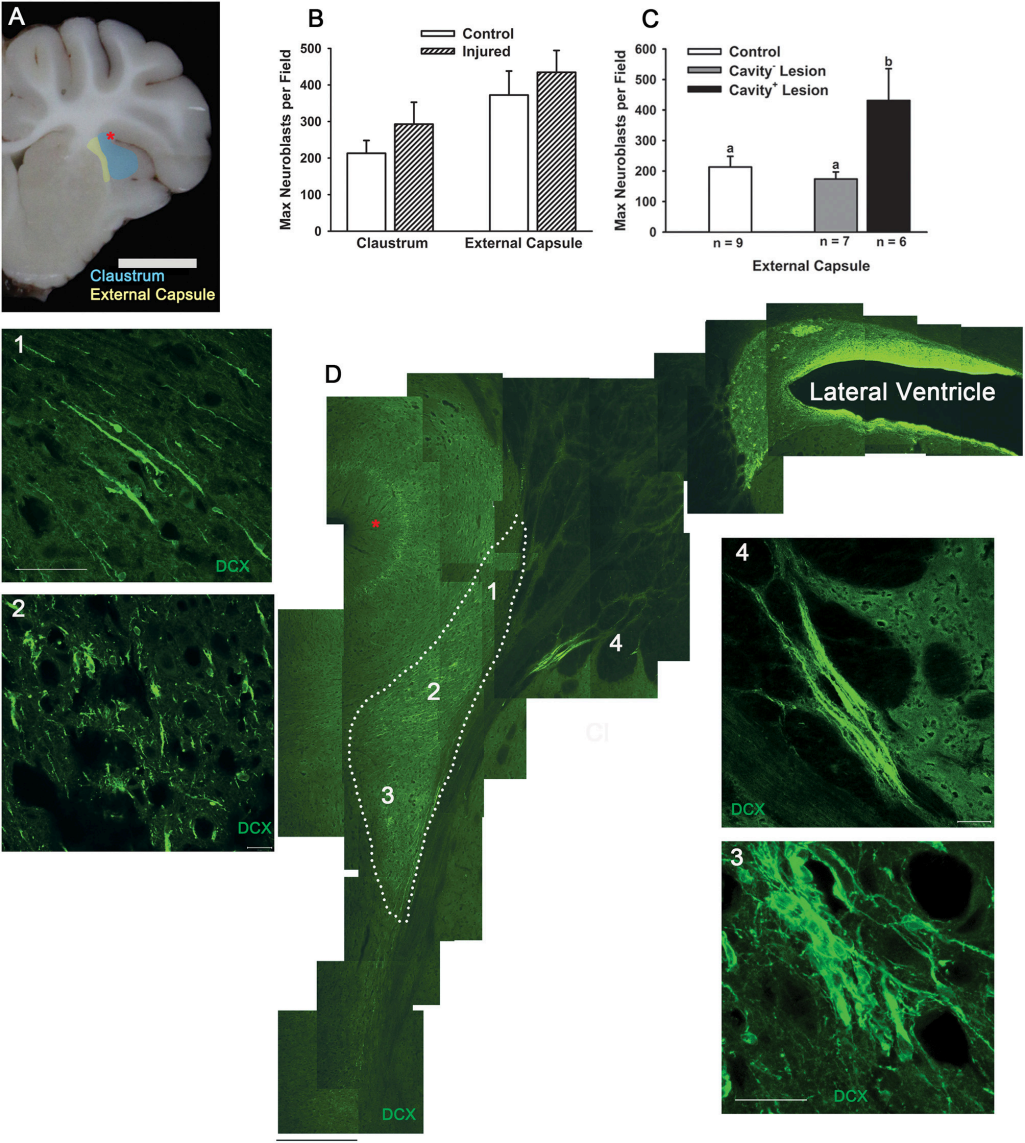
**Cortical impact does not reduce the number of neuroblasts in the claustrum**. We observed large chains of neuroblasts traveling from the SVZ through the external capsule and entering the claustrum in sham piglets. We compared the density of neuroblasts in the external capsule and claustrum in piglets injured or receiving sham surgery at PND 7 and brains collected at PND 14. **(A)** Neuroblasts were quantified in the white matter (yellow) ventral to the SVZ and in the white matter adjacent to the claustrum (Cl, blue). **(B)** The maximum density of neuroblasts in the external capsule and claustrum in injured piglets were not different from sham piglets. In injured piglets, the density of neuroblasts in the claustrum was nearly three-fold greater than in the injured gray matter of the rostral gyrus indicating that postnatal population of this region is still prioritized after TBI in the gyrencephalic brain. **(C)** When piglets were binned according to lesion type, the neuroblast density was greater in the external capsule in piglets with “cavity^+^ lesions” than shams or “cavity^−^ lesions” (^a, b^Means ± SEM with different letters differ, *P* < 0.05). **(D)** Low power view of the SVZ, ventral white matter, external capsule, and claustrum (Cl, indicated with broken line) with neuroblasts detected with DCX (green) with the red star corresponding to the sulci marked with a red star in **(A)**. **(D1)** In the dorsal claustrum, long and thick chains of neuroblasts are observed in a migratory morphology. **(D2,D3)** In the ventral claustrum, neuroblasts are extremely dense with individual neuroblasts and various sizes of clusters. **(D4)** A large cluster of neuroblasts approximately 1000 μm in length appears to migrate through the external capsule. Scale bars: **(A)** = 1 cm; **(D)** = 200 μm; **(D4)** = 100 μm; **(D1–D3)** = 20 μm.

As TBI during childhood is superimposed upon ongoing development, we investigated the effect of TBI on an actively postnatally-populating region in the piglet. In coronal sections of sham or injured piglets, large chains of neuroblasts of a migratory morphology appeared to be exiting the ventral SVZ into the external capsule and then a portion were observed to enter the claustrum (Figures 5A,D). Within the claustrum, neuroblast morphology varied according to region. In the dorsal claustrum, neuroblasts were observed in long, narrow chains (Figure 5D1). In the central claustrum, individual neuroblasts were present in either a differentiating or a migratory morphology and chains were absent (Figure 5D2). In the ventral claustrum, a mix of large chains of neuroblasts and individual migratory or neuroblasts with a differentiating morphology was observed (Figure 5D3). The density of neuroblasts in the external capsule and in the claustrum was not different in response to injury (Figure 5B), but the maximum density of neuroblasts was greater in the external capsule in cavity^+^ lesions than sham or cavity^−^ lesions (*P* < 0.05; Figure 5C). The density of neuroblasts in the claustrum was two-fold denser than in injured rostral gyrus highlighting the apparent prioritization of the immature brain to continue with development.

## Discussion

This is the first study to determine the effect of cortical impact on the distribution of neuroblasts in the white matter path to the injury, at the injury, and at an actively postnatally-populating region in any species. Various models of injury and stroke in adult and immature rodents have shown that neuroblasts migrate from the SVZ to striatum or cortex where they replace, at least temporarily, injured neurons (Gordon et al., 2007; Li et al., 2008; Jin et al., 2010; Saha et al., 2013). However, the cortex of rodents, which lacks gyri and gyral white matter that may complicate repair efforts after TBI, may not accurately model the mechanisms of injury and repair in children; additionally, models thus far of TBI do not use an impact event to cause the injury but aspiration or freezing (Sundholm-Peters et al., 2005; Covey et al., 2010; Saha et al., 2013). We have previously described ectopic neurons in the damaged white matter of a child after TBI in a region excised for extra-temporal epilepsy and hypothesized that neuroblasts traveling to the injured gyrencephalic brain may result in epileptic foci (Taylor et al., 2013). An additional incidental finding was a clinically silent infarct in the subcortical white matter in a 1 month old infant who died of sepsis where an increased density of neuroblasts was observed around the infarct (Taylor et al., 2013). We hypothesize that neurogenesis stimulated by TBI and other causes of focal brain damage in the gyrencephalic brain may contribute to post-traumatic sequelae in addition to injury repair, in contrast to the predominant hypothesis in the TBI literature that neuroblasts target the injury to aid in successful repair without detriment.

In our model where both the gray matter and white matter of the rostral gyrus are damaged from cortical impact, we found a greater density of neuroblasts in gyral gray matter compared to sham piglets, but only in lesions that were cavitated. Within cavity^+^ lesions, neuroblasts were most dense at the ventral portion of the gyri and were noticeably absent in dorsal portions of the gyri appearing to abandon attempts at salvage. It is difficult to compare the neuroblast distribution in gray matter in our model to the response to a cortical lesion from to aspiration or cryoinjury in the lissencephalic brain as these papers only describe the ventral or ventral and lateral portion of the lesion, which demonstrates increased neuroblast density in the gray matter in immature (Covey et al., 2010) and adult mice (Saha et al., 2013), but does not describe the dorsal portions of the lesion. One paper in adult mice reported an absence of neuroblasts in the gray matter adjacent to a lesion when the aspiration lesion did not have gray matter present between the lesion cavity and corpus collosum (Sundholm-Peters et al., 2005). In rodent studies, the low variability of lesion size after injury to the cortex is due to either biological uniformity specific to this species, due to the exclusion of variable lesions, or that these models of TBI do not use a cortical impact (Covey et al., 2010; Saha et al., 2013). Certainly, an impact model of TBI creates a complicated and cluttered tissue response. Increased neuroblast density at the lesion may be achieved by, (1) re-direction and concentration of neuroblasts to the ventral portion of the gyrus, which has more potential for salvage and repair then the damaged dorsal portions of the gyrus, (2) apoptosis and/or necrosis of neuroblasts in the dorsal portion, (3) decreased binding of antibodies the dorsal injured gyri, or (4) closer proximity to the SVZ (Covey et al., 2010; Saha et al., 2013; Costine et al., 2015). In this experiment, we have not determined if the neuroblasts are specifically targeting the cavity or, conversely, if neuroblasts migrating along gyral white matter tracts are collecting in the white matter creating a bottleneck of neuroblasts. It is possible that a longer piglet survival time might reveal a greater number of neuroblasts in the dorsal regions of the injured gyri. We hypothesized that damaged gyral white matter tracts may impede neuroblasts from reaching damaged gyral gray matter. Indeed, there were a lower number of BrdU^+^ neuroblasts in the white matter leading from the SVZ to the rostral gyrus ipsilateral to injury in injured vs. sham piglets (Figure 1D). It is conceivable that the burr hole and dural manipulation stimulates the release of cytokines that may attract neuroblasts in surgical sham subjects, but the number of neuroblasts responding to the cytokines may be impeded by white matter damage in injured subjects (Tang et al., 2014). In large brained species, the distance of the diffusion of the cytokines from the injury relative to the size of the brain may limit the distance where neuroblasts may be actively recruited.

An additional finding at the injury site was the apparent differentiation of neuroblasts in damaged white matter. Within cavity^+^ lesions, neuroblasts were densely arranged adjacent to a portion of the lesion cavity edge in the ventral portion of the gyri. This is similar to work in rodents revealing differentiating neuroblasts in the gray matter lesion (Covey et al., 2010; Saha et al., 2013), but shifted to the white matter in this gyrencephalic species. In the dense network of neuroblasts in the white matter, a large proportion of neuroblasts were observed to have several neurites. To further investigate these neuroblasts, we probed for calretinin, an early marker of differentiation; calretinin is a calcium-binding protein expressed in GABAergic neurons including a subset of interstitial neurons (Suárez-Solá et al., 2009; Wu and Sun, 2015). Calretinin^+^ cells with neuronal morphology were located throughout the white matter of injured and sham piglets. In injured piglets, some calretinin^+^/DCX^+^ neurons and NeuN^+^/calretinin^−^ neurons were in the white matter. This population may be resident interstitial cells or newly differentiated neurons in the white matter in response to injury. In the gyrencephalic brain, interstitial neurons mediate brain development and persist into adulthood in the gyral white matter; this population is absent in rodents (Suárez-Solá et al., 2009). Future studies will aim to quantify neurons in the white matter at different time points after injury as well as further characterize the injury environment and these potentially differentiating neuroblasts. While the migration of neuroblasts in the corpus callosum has been examined after TBI, neuroblasts with a differentiating morphology in the white matter have not yet been described after TBI in any species (Sundholm-Peters et al., 2005; Covey et al., 2010; Saha et al., 2013). The only phenomena similar to what we describe here is in humans where neuroblasts localized to a white matter infarct in an infant (Taylor et al., 2013) and around chronic white matter lesions in adult multiple sclerosis patients (Chang et al., 2008). Focal differentiation of neuroblasts around white matter lesions post-TBI in the human brain may be an attempt at a “quick fix” with long-lasting effects.

The role of potentially differentiating neuroblasts in the white matter after cortical impact is not known. One possibility is a recapitulation of development as calretinin^+^ interstitial neurons are crucial to development (Suárez-Solá et al., 2009; Wu and Sun, 2015). Vertical plasticity has been described in human children with hydranencephaly where the children developed functions ascribed to the neocortex in the near-complete absence of a neocortex (Shewmon et al., 1999). These children were born with scarce neocortex and lacked neocortical functions as infants and toddlers, but developed neocortical functions during childhood. The neurologists treating these children proposed that the functions ascribed to the cortex developed subcortically (Shewmon et al., 1999). This population of differentiating neuroblasts may be transient and temporarily supply growth factors to the injured white matter and then regress. Alternatively, ectopic neurons that persist may develop into direct epileptic foci or indirectly through influencing surrounding circuits (Petit et al., 2014). Though neurons generated from the SVZ in healthy subjects are GABAergic, the possibility of neurons from the SVZ serving as epileptic foci is supported by work in stroke models where SVZ-derived differentiated neurons in the cortex generated excitatory post-synaptic currents (Lai et al., 2008). Furthermore, GABA is depolarizing in the immature brain and GABAergic neurons revert back being excitatory after injury, which may be a recapitulation of development (van den Pol et al., 1996; Wu and Sun, 2015). Repair mechanisms may have both beneficial and deleterious long-term effects.

Of the neuroblasts at the injury site, less than 1% were born (BrdU^+^) in the 2 days before or the 2 days after injury. This low percentage is similar to our previous work estimating the total number of neuroblasts in the entire white matter in sham or injured piglets (Costine et al., 2015). There was no effect of injury on the number of BrdU^+^ neuroblasts in the white matter in the current experiment (the white matter tract leading from the SVZ to the injury) or our past experiment (all white matter). However, we did find an increased number of BrdU^+^ neuroblasts at the lesion site in the gray matter though they comprised a very small portion of total neuroblasts. We can conclude that the majority of neuroblasts that arrived at the lesion were born prior to PND 4, and likely were born prior to birth. An increase in neuroblast density at the lesion was largely achieved without stimulation of cell division to create new neuroblasts, which is similar to work in immature rodents where only 10% of neuroblasts were BrdU^+^ when BrdU was administered for 4 days post-injury (Covey et al., 2010). The low percentage of neuroblasts labeled with BrdU reported here and reported in immature rodents (Covey et al., 2010) is in contrast to adult rodents (Sundholm-Peters et al., 2005). In uninjured adult mice, the number of BrdU^+^ neuroblasts in the corpus callosum were doubled in response to injury to the cortex (Sundholm-Peters et al., 2005). In the early postnatal brain where an abundance of neuroblasts are migrating throughout the white matter, a further increase in the production of neuroblasts to support lesion repair may not be possible.

In gyrencephalic species where the white matter is abundant, the number of neuroblasts in the white matter does not predict the number of neuroblasts collected at the lesion site. In our unbiased quantification of neuroblasts in the white matter from the SVZ leading to the injured rostral gyrus including the corpus callosum, centrum semiovale, and gyral white matter, there was no effect of cortical impact on the number of neuroblasts though we found an increase in the density of neuroblasts at the lesion site. In the uninjured PND 14 piglet, we have previously demonstrated large chains of neuroblasts moving rostrally in the rostral migratory stream, and demonstrate a ventrolateral migratory stream in our current work (Costine et al., 2015). In addition to these major pathways, all white matter of the neocortex contained individual or small chains of neuroblasts with a migratory morphology (Costine et al., 2015). The persistence of doublecortin protein in differentiated neurons in cortical layer II, which was damaged after cortical impact, is similar to other higher mammals, has been demonstrated to persist into adulthood, and is hypothesized to impart increased complexity through continued plasticity of GABAergic interneurons in higher mammals, but does not persist through adulthood in rodent species (Cai et al., 2009; Radonjic et al., 2014). In contrast, neuroblasts produced in the SVZ in healthy postnatal rodents fail to enter the neocortex after the regression of radial glia possibly due to the absence of white matter tracts entering the neocortex (Brazel, 2003). Any postnatal population of the neocortex in the Yorkshire/Landrace piglet may impart plasticity, but does not increase the total number of neurons as neocortical neuron number does not increase from birth to adulthood in piglets, similar to humans (Jelsing, 2006). The immature gyrencephalic brain appears to use white matter tracts in the neocortex as highways for postnatal neocortex development, and to continue the analogy—the population of cars on a highway (white matter) to a city cannot be used to estimate the population of the city (lesion site).

We sought to determine if the developing gyrencephalic brain prioritized development over injury repair or vice versa. The postnatal population of the olfactory bulb via the rostral migratory stream is the most studied among species including rodents, piglets, and humans (Sundholm-Peters et al., 2005; Sanai et al., 2011; Costine et al., 2015). Neuroblasts also migrate from the SVZ into the ventral migratory stream (Sanai et al., 2011) and have been found to populate the Islands of Calleja in rodents (De Marchis et al., 2004). Here, we observed a large population of neuroblasts moving from the SVZ into a ventral migratory stream and into the claustrum. Postnatal development of the claustrum has been described in the rat, rabbit, and cat, and the specific role of migrating neuroblasts populating the claustrum up to PND 4 was hypothesized to occur to explain the late differentiation of interneurons (Adinolfi and Levine, 1986; Wójcik et al., 2002; Kowianski et al., 2008). Here, we demonstrate this predicted postnatal population of interneurons in the claustrum of piglets at PND 14, which is later than observed in rodents, perhaps allows greater plasticity to this brain region that is important in cross-modal sensory integration. After TBI, the density of neuroblasts in the claustrum was not diminished. This is similar to adult rodents where migration to the olfactory bulb was not diminished even with an increased number of neuroblasts migrating dorsally to the cortical lesion (Sundholm-Peters et al., 2005). Most of the neuroblasts in the claustrum and gray matter of the rostral gyrus were born prior to PND 5 as BrdU^+^ neuroblasts were rare in both regions when BrdU was administered as early as PND 5. Though the density of neuroblasts in the claustrum was not altered from TBI, the density of neuroblasts in the external capsule was greater in subjects with cavity^+^ lesions. In the immature piglet brain, it appears that cortical impact may induce neuroblasts to concentrate in lesions with a cavity without compromising development of brain regions, and may potentially result in greater postnatal population of other brain regions.

In conclusion, cortical impact to the rostral gyrus of the immature piglet brain increased the density of neuroblasts in the gray matter and unexpectedly caused focal densities of neuroblasts in the injured white matter. Consistent with other models of injury in immature animals, injury did not stimulate de novo neurons. However, unlike TBI models in rodents, both the lesion pattern and neuroblast distribution in the lesion cortex were heterogeneous. Neuroblast density was increased in the gray matter, but this increase was in cavitated lesions only. In the white matter, neuroblasts aligned along the edge of the cavity and had a differentiating morphology. This meshwork of potentially differentiating neuroblasts may become interstitial neurons, may develop into new cortex via vertical plasticity, may be support the re-building of white matter recapitulating development, may support repair temporarily and regress, or may ultimately develop into epileptic foci responsible for some of the long-term effects of TBI. Immature gyrencephalic animals can serve as a platform to study TBI in infants and children where white matter, which is preferentially vulnerable after TBI, may complicate the repair response. Future work will focus on the interplay of neuroblasts and interstitial neurons in white matter repair, the potential for this remodeled white matter to create post-traumatic epileptic foci, to determine if postnatal population of brain regions is affected, and to examine the potential effect of age on these processes during immaturity. If it is determined that neuroblast differentiation impedes white matter recovery in the gyrencephalic brain, then modulation and even repression of the targeting of neuroblasts to white matter injury may improve recovery in infants and children after TBI.

## Author contributions

BC, ST, and AD designed the experiments. ST, KK, and CD performed the animal experiments. ST, BC, CS, and KK performed the immunohistochemistry and quantification. BC, AD, ST, and DM analyzed and interpreted the data. BC and ST drafted the paper. All authors critically reviewed, revised, and approved the final version of the paper.

### Conflict of interest statement

The authors declare that the research was conducted in the absence of any commercial or financial relationships that could be construed as a potential conflict of interest. The reviewer EB and handling Editor declared that even though they share the institution they work at different departments, and the handling Editor states that the process met the standards of a fair and objective review.
